# Equity and Noncommunicable Disease Reduction under the Sustainable Development Goals

**DOI:** 10.1371/journal.pmed.1001872

**Published:** 2015-09-08

**Authors:** Harald Schmidt, Anne Barnhill

**Affiliations:** 1 Department of Medical Ethics and Health Policy, Perelman School of Medicine, University of Pennsylvania, Philadelphia, Pennsylvania, United States of America; 2 Center for Health Incentives and Behavioral Economics, Perelman School of Medicine, University of Pennsylvania, Philadelphia, Pennsylvania, United States of America

## Abstract

Harald Schmidt and Anne Barnhill highlight the need to ensure equity in the proposed Sustainable Development Goals.

**Summary Points**

- Currently proposed Sustainable Development Goals (SDGs) include a timely call to significantly reduce the burden of noncommunicable diseases (NCDs).
- Existing policy guidance highlights cost-effective interventions for NCDs, but focusing just on cost-effectiveness risks exacerbating socioeconomic and health inequalities rather than reducing them.
- In implementing the SDGs, targets and interventions that benefit the worst off should be prioritized.
- The United Nations should develop practical guidance to assist policy makers at the country level with incorporating equity considerations.

## Introduction

Healthy life expectancy at birth in Sierra Leone is 46 years. In Japan, it is 84 years [1]. The UN Millennium Development Goals (MDGs) set out ambitious objectives to reduce such and further inequalities. Despite criticism, the MDGs are widely praised for having galvanized national and international development efforts in unprecedented ways [2]. Currently proposed successor Sustainable Development Goals (SDGs) seek to address newly emerged policy issues and include a call to significantly reduce the burden of noncommunicable diseases (NCDs). NCDs directly impact health inequality and poverty [1]. Their recognition is timely and to be welcomed categorically. However, ambiguity in the SDGs’ current guidance risks that states’ efforts to reduce NCDs exacerbate socioeconomic and health inequalities, rather than reduce them. We urge that more attention needs to be given to improving the situation of the worst off and make three concrete proposals towards this end.

## UN Guidance

The UN General Assembly's (GA) Open Working Group on Sustainable Development Goals recently submitted a proposal for SDGs (Box 1) [3]. The SDGs’ third goal concerns health and comprises 13 targets, including that states reduce “by one-third premature mortality from non-communicable diseases” by 2030.

**Box 1. Goal 3 of the Proposed SDGs: “Ensure Healthy Lives and Promote Well-Being for All at All Ages”**

The proposed SDGs have been formulated by the UN to succeed the MDGs and are expected to be adopted in its 2015–2016 session. The SDGs comprise 17 goals that include targets in relation to poverty, nutrition, health, education, gender equality, employment, energy, climate change, and global partnerships. The health goal has 13 targets as set out below (note that the numbering is not explicitly intended to indicate priority):

3.1 By 2030 reduce the global maternal mortality ratio to less than 70 per 100,000 live births.
3.2 By 2030 end preventable deaths of newborns and of children under five.
3.3 By 2030 end the epidemics of AIDS, tuberculosis, malaria, and neglected tropical diseases and combat hepatitis, water-borne diseases, and other communicable diseases.
3.4 By 2030 reduce by one-third premature mortality from non-communicable diseases (NCDs) through prevention and treatment and promote mental health and well-being.
3.5 Strengthen prevention and treatment of substance abuse, including narcotic drug abuse and harmful use of alcohol.
3.6 By 2020 halve global deaths and injuries from road traffic accidents.
3.7 By 2030 ensure universal access to sexual and reproductive health care services, including for family planning, information and education, and the integration of reproductive health into national strategies and programmes.
3.8 Achieve universal health coverage (UHC), including financial risk protection, access to quality essential health care services, and access to safe, effective, quality, and affordable essential medicines and vaccines for all.
3.9 By 2030 substantially reduce the number of deaths and illnesses from hazardous chemicals and air, water, and soil pollution and contamination.
3.a Strengthen implementation of the Framework Convention on Tobacco Control in all countries as appropriate.
3.b Support research and development of vaccines and medicines for the communicable and non-communicable diseases that primarily affect developing countries, provide access to affordable essential medicines and vaccines in accordance with the Doha Declaration, which affirms the right of developing countries to use to the full the provisions in the TRIPS [Trade-Related Aspects of Intellectual Property Rights] agreement regarding flexibilities to protect public health, and, in particular, provide access to medicines for all.
3.c Increase substantially health financing and the recruitment, development and training, and retention of the health workforce in developing countries, especially in LDCs [least developed countries] and SIDS [small island developing states].
3.d Strengthen the capacity of all countries, particularly developing countries, for early warning, risk reduction, and management of national and global health risks.

Available: https://sustainabledevelopment.un.org/topics/sustainabledevelopmentgoals (accessed June 9, 2015)

The importance of NCDs has already been recognized in several earlier key UN documents. Its 2011 High-Level Meeting on the Prevention and Control of NCDs resulted in a political declaration (NCD Declaration), adopted subsequently by the GA [4]. The declaration characterized NCDs as a threat to development and a cause and consequence of poverty and inequality. It emphasized the importance of several established initiatives, including full implementation of the World Health Organization’s (WHO) Framework Convention on Tobacco Control (FCTC). As a follow-on to the NCD Declaration, the WHO’s Global Action Plan for the Prevention and Control of Noncommunicable Diseases 2013–2020 (NCD Plan) set out nine voluntary global targets for NCDs, including the goal of a 25% mortality reduction for key chronic conditions [5].

The NCD Plan identified 81 policy options, highlighting 14 as “very cost-effective and affordable for all countries” (Box 2). The introduction to these policies notes that alongside factors such as effectiveness, cost-effectiveness, and implementation capacity, “impact on health equity” needs to be considered—yet, there is no concrete guidance on how this might be done [5]. An informal 2013 WHO note on progress with achieving the NCD Declaration’s goals recommended implementation of the NCD Plan’s “very cost-effective and affordable interventions” but made no mention of equity [6]. The recent draft SDG goals call on states “to reduce inequalities within and among countries” (goal 10) and to “ensure equal opportunity and reduce inequalities of outcome” (target 10.3) but provide no guidance on what this means for health policy.

**Box 2. Interventions Identified as Very Cost-effective and Affordable Interventions, WHO Global Action Plan for the Prevention and Control of Noncommunicable Diseases**

**Tobacco Use**

- Reduce affordability of tobacco products by increasing tobacco excise taxes
- Create by law completely smoke-free environments in all indoor workplaces, public places, and public transport
- Warn people of the dangers of tobacco and tobacco smoke through effective health warnings and mass media campaigns
- Ban all forms of tobacco advertising, promotion, and sponsorship

**Harmful Use of Alcohol**

- Regulating commercial and public availability of alcohol
- Restricting or banning alcohol advertising and promotions
- Using pricing policies such as excise tax increases on alcoholic beverages

**Unhealthy Diet and Physical Activity**

- Reduce salt intake
- Replace trans fats with unsaturated fats
- Implement public awareness programmes on diet and physical activity

**Cardiovascular Disease and Diabetes**

- Drug therapy (including glycaemic control for diabetes mellitus and control of hypertension using a total risk approach) and counselling to individuals who have had a heart attack or stroke and to persons with high risk (≥ 30%) of a fatal and nonfatal cardiovascular event in the next 10 years
- Acetylsalicylic acid for acute myocardial infarction

**Cancer**

- Prevention of liver cancer through hepatitis B immunization
- Prevention of cervical cancer through screening (visual inspection with acetic acid [VIA] or Pap smear [cervical cytology], if very cost-effective), linked with timely treatment of precancerous lesions

Available: http://www.who.int/nmh/events/ncd_action_plan/en/ (accessed June 9, 2015)

## Problems

The scant guidance on equity raises concern, as does the emphasis on cost-effectiveness: reducing the burden of NCDs cost-effectively is not the same as reducing it equitably. In particular, interventions that cost-effectively reduce overall NCD burden can preferentially benefit better-off groups and widen inequalities [2,7]. But the most morally urgent demand is to help the worst off [2,8].

The signaling effect of high-level policy such as the SDGs is considerable and can directly impact policy development at the country level. Moreover, there can be pressure from donors and supranational organizations to tie funding to progress towards goals such as those set out in the SDGs. This can lead to a disproportionate focus on overall NCD mortality reduction, without sufficient attention paid to the distribution of benefits across groups.

Which groups benefit to what extent matters ethically [7]. In goal 1, the SDGs call particular attention to those who are worst off economically: “[e]nd poverty in all its forms… by 2030, eradicate extreme poverty for all people everywhere, currently measured as people living on less than [US]$1.25 a day.” For the present purposes, it is adequate to understand the worst off as centrally those with the lowest income, even if attention should also be paid to distributions across racial, ethnic, gender, educational, and geographic (rural versus urban) groups [2,7].

Prioritizing the worst off requires targeting risk factors and diseases that disproportionately affect them and implementing interventions that reach and benefit them [7]. Evidence from high-income countries suggests that NCD rates are higher in disadvantaged and marginalized communities, but low-income and middle-income countries show a more complex pattern. There are higher rates of some risk factors, such as tobacco use, in disadvantaged groups but lower rates of other risk factors [8].

The SDGs and the NCD Plan’s list of 14 “very cost-effective and affordable” interventions both include several interventions that are likely to benefit worst-off groups, including creating smoke-free workplaces; banning tobacco advertising, promotion, and sponsorship; increasing excise taxes on tobacco; and regulating the availability of alcohol [9,10]. However, there are also interventions from which the benefits are likely to be distributed differently. Consider, for example, the NCD Plan’s recommendation to implement breast cancer screening. If uptake of screenings increases as intended, health inequalities both within and between countries likely will narrow. Yet, mammography screening only helps populations with access to the health system and, among them, favors the more health literate [2]. The same is likely to be true of the NCD Plan’s recommendation to increase access to diabetes and hypertension drug therapy and counseling for heart attack or stroke patients. By contrast, broader public health measures—such as banning tobacco use in public places—that do not require education, resources, or regular health care system access benefit worse-off groups more directly.

The principal problem with the SDGs’ vague guidance to “ensure equal opportunity and reduce inequalities of outcome” in combination with the target of reducing overall mortality is that it could sanction reducing NCDs by targeting preferentially easier to reach populations—repeating and continuing a trend that has already been identified regarding the MGDs [2,7]. This approach would conflict with the overall spirit of the SDGs, which calls, in goal 1, to eradicate extreme poverty.

## Moving Forward

To give meaningful substance to the SDGs’ call to reduce inequalities, there needs to be an unambiguous acknowledgment that pursuing cost-effective interventions and achieving overall NCD mortality reductions is insufficient. The urgent moral demand in terms of promoting equity is to improve the situation of the worst off [7]. Three concrete measures can help make progress towards this end.

### Clarity in SDG Wording

Target 3.4 could be revised to read something like: “by 2030 reduce by one-third premature mortality from NCDs through prevention and treatment and promote mental health and well-being, *with particular emphasis on improving the status of the worst off*” (suggested addition in italics). Clearly, the wording of high-level guidance alone cannot sustainably steer practice, but without a strong and unambiguous SDG vision, there is a real risk that genuine equity considerations in reducing NCDs will not be a firm part of the horizon that must guide our navigation.

### Prioritizing Targets

In roadmapping action relating to the 13 discrete targets under the SDGs’ goal 3, states should ensure that those targets that are most likely to benefit worse-off groups are prioritized as far as possible, particularly targets 1, 2, 5, 6, 9, and 3a (see Box 1). The same goes for states implementing the NCD Plan.

To be clear, how policy makers prioritize interventions should also take into account total cost and cost-effectiveness. Much depends upon a country’s level of development and the capacities of the health care and public health system [2]. We are not arguing here for a specific view about how much priority the worst off should be given. Rather, we want to emphasize that there can be trade-offs between equity and cost-effectiveness and that cost-effectiveness alone should not be decisive. The dominant focus on cost-effectiveness may obscure this point, particularly when emphasis is placed on reducing overall NCD levels. Our call to prioritize interventions that benefit the worst off is intended to encourage policy makers to reflect more actively on the distribution of benefits across population groups in considering which of the SDGs’ goals and targets to pursue with what level of intensity.

### Equity Guidance

To assist in the process, there needs to be concrete guidance on how to select interventions that genuinely promote equity [7,11]. A helpful step has been made with a recent WHO report on equity and UHC [12]. The report focuses centrally on justifying priority for the worst off and takes a broad understanding of UHC, encompassing not just clinical but also public health services. Yet, its discussion is overwhelmingly centered on clinical services, and the report is targeted specifically at policy makers in health ministries. In contrast, equitable NCD policy calls for a broader set of interventions than those coordinated by health ministries and requires concerted intersectoral action across different government departments, civil society, and other actors.

The UN should therefore establish a working group to produce complementary practical guidance that would identify policy approaches that integrate equity, showcasing concrete examples. This guidance would include an inventory of key policy options that have been shown to be effective in reaching underserved or otherwise disadvantaged groups and practical recommendations on how meaningful equity indicators can be measured and incorporated into planning, implementing, and evaluating NCD policies [13,14]. The guidance should also address salient fairness issues that can arise from targeting specific populations. Tobacco-, alcohol-, and overweight-related NCDs have a strong behavioral component. Penalizing personal responsibility policies, rather than appropriate action at the level of the social determinants of health, can be tempting for policy makers [15]. The guidance should set out the scope and limitations of policies focused on personal responsibility. Furthermore, even if done benevolently, prioritizing some groups can appear, or be, stigmatizing [16]. Outlining strategies for avoiding real or perceived stigma will be of additional benefit.

## Conclusion

The SDGs rightly call for action to reduce NCD-associated morbidity and mortality, but they do not offer guidance on how to achieve this reduction equitably and fairly, nor is such guidance found in other key policy, such as the NCD Plan. It is imperative that the SDGs clearly express that equity requires preferentially helping the worst off. Prioritizing among the targets of the SDGs’ goal 3 accordingly and providing further concrete guidance can assist with translating the SDGs’ aspirations into practice. Ideally, this will help improve the health and welfare of those who are the worst off.

## Abbreviations

FCTC: Framework Convention on Tobacco Control
GA: General Assembly
LDC: least developed country
MDG: Millennium Development Goal
NCD: noncommunicable disease
SDG: Sustainable Development Goal
SIDS: small island developing states
TRIPS: Trade-Related Aspects of Intellectual Property Rights
UHC: universal health coverage
VIA: visual inspection with acetic acid
WHO: World Health Organization
